# Cognitive and Emotional Resilience Seven Years After Brain Tumor Resection

**DOI:** 10.7759/cureus.104385

**Published:** 2026-02-27

**Authors:** Abigail Kellar, Ian Moore, Evelyn Embry, Kalpana P Padala, Prasad R Padala

**Affiliations:** 1 Geriatric Research Education and Clinical Center (GRECC), Central Arkansas Veterans Healthcare System, Little Rock, USA

**Keywords:** central nervous system tumor, cognitive testing, depression, depression in chronic illness, falcotentorial meningioma, left parasellar meningioma, memory disorders clinic, meningioma, mild cognitive impairment (mci), neurocognitive impairment

## Abstract

Meningiomas are common and are associated with physical, cognitive, and emotional changes due to their growth and treatment methods. Longterm cognitive recovery following surgery is often underexplored. In this case report, we highlight resiliency and recovery following brain tumor resection and emphasize neuropsychology and psychiatry's role in long-term care. We present the case of a 64-year-old Black female Veteran followed with serial neuropsychological evaluations up to seven years after her temporal craniotomy and parasellar meningioma resection. Initial evaluations showed cognitive deficits and emotional instability, leading to a diagnosis of mild cognitive impairment (MCI). However, she continued to improve, and in the most recent evaluation, she was deemed to be of normal cognition, although depressive symptoms persisted. This case demonstrates significant cognitive recovery post-meningioma resection, underscoring the brain's resilience and the critical role of the neuropsychology and psychiatry services in long-term care and recovery.

## Introduction

Meningiomas are the most common type of primary central nervous system tumor. As brain tumors continue to grow and displace brain tissue, patients may experience changes in their physical, cognitive, or emotional functioning; the nature and extent of which are often dependent on the size and location of the tumor as well as the methods used to treat it [1].

Currently, transcranial resection, stereotactic radiosurgery, and proton beam therapy are amongst the most common and effective treatments for parasellar meningiomas; however, they all come with the risk of potential treatment-related cognitive, emotional, and functional decline. Postoperatively, verbal deficits and working memory deterioration have been more pronounced in patients with a left-sided meningioma. Patients’ rapid cognitive and functional recovery takes place in the first three to six months following resection and drastically slows one year post operation [1]. Regarding the impacts of non-surgical interventions, studies have shown that 50-90% of adults with a brain tumor reported experiencing cognitive dysfunction for at least six months following fractionated partial or whole-brain radiation [2,3]. Specifically, patients most commonly experienced impairments in verbal and spatial memory, attention, and novel problem-solving ability, with their deficits becoming increasingly pronounced the longer treatments lasted [3].

Meningiomas and their associated treatments also have adverse impacts on mood and personality functioning. For example, postoperative subjective functioning, anxiety, and depression have been found to worsen in meningioma patients [4-6]. Commonly, geriatric psychiatry and neuropsychology services are involved with the diagnosis and management of neurodegenerative conditions, where patient prognosis is typically poor, and treatment options are generally focused on symptom management and supportive interventions [7,8]. Even in cases where protracted recovery is possible post insult (e.g., traumatic brain injury (TBI), cerebrovascular accidents (CVA), brain surgery, etc.), psychiatry and neuropsychology may only be involved in acute and post-acute care but not long-term recovery.

However, psychiatry and neuropsychology services can assist with patients' long-term care via serial evaluations to help create and modify long-term treatment plans to address patients' changing cognitive, emotional, and functional needs. Data on preoperative and postoperative quality of life are scarce, highlighting the importance of the current report. To illustrate the importance of neuropsychology and psychiatry services to patients' long-term medical and mental healthcare, we present a case report of a patient with repeat neuropsychological testing following a parasellar meningioma resection to illustrate the potential for improvements in cognitive, emotional, and functional changes post treatment.

## Case presentation

A 64-year-old single Black female Veteran with 14 years of education, presented to a memory disorders clinic in 2023 for cognitive testing due to ongoing cognitive and emotional difficulties following a left temporal craniotomy and complete resection of a left parasellar meningioma in 2016. 

**Figure 1:**
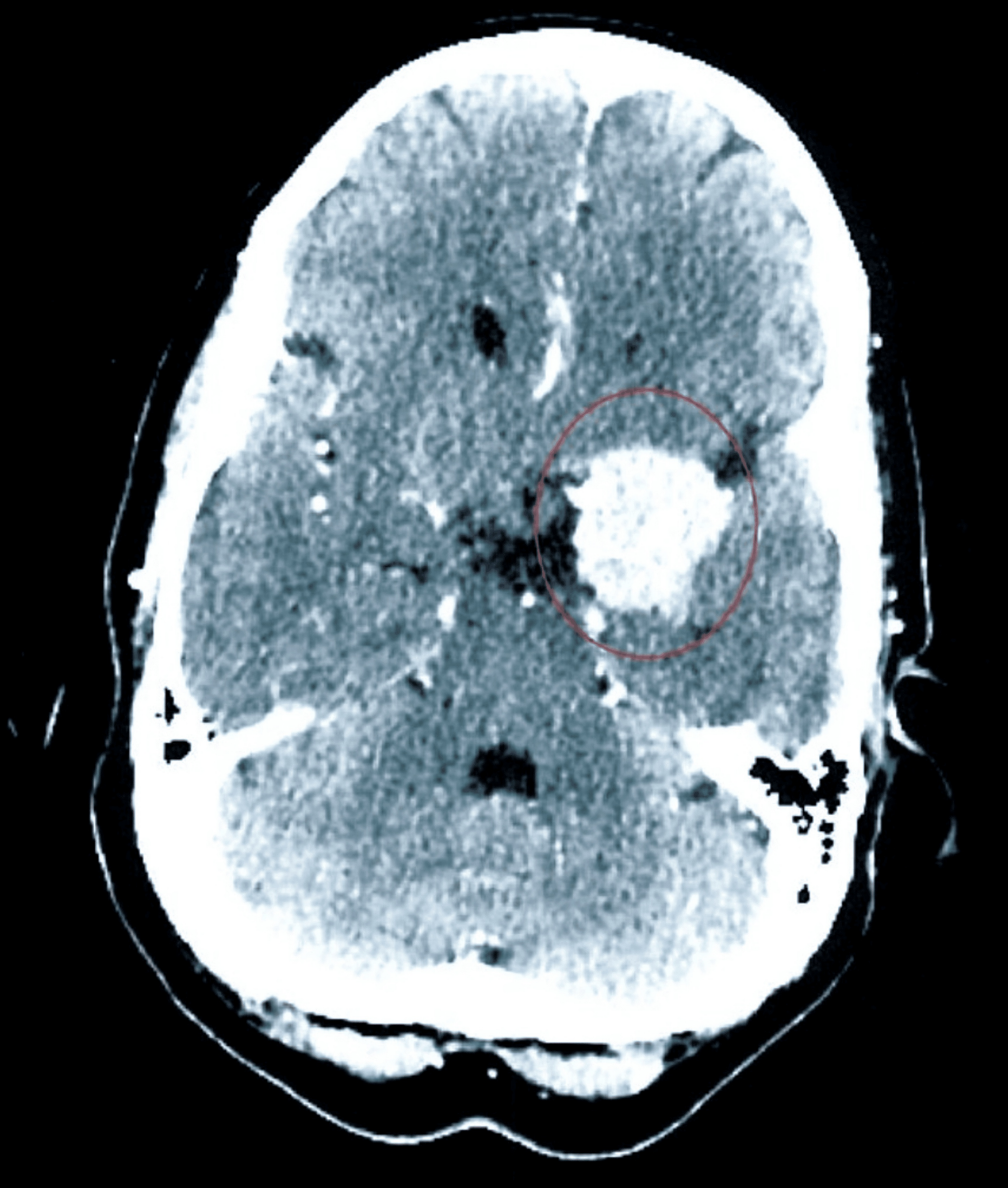
Pre-resection CT scan from October 2015 showing the tumor in the circled area

**Figure 2:**
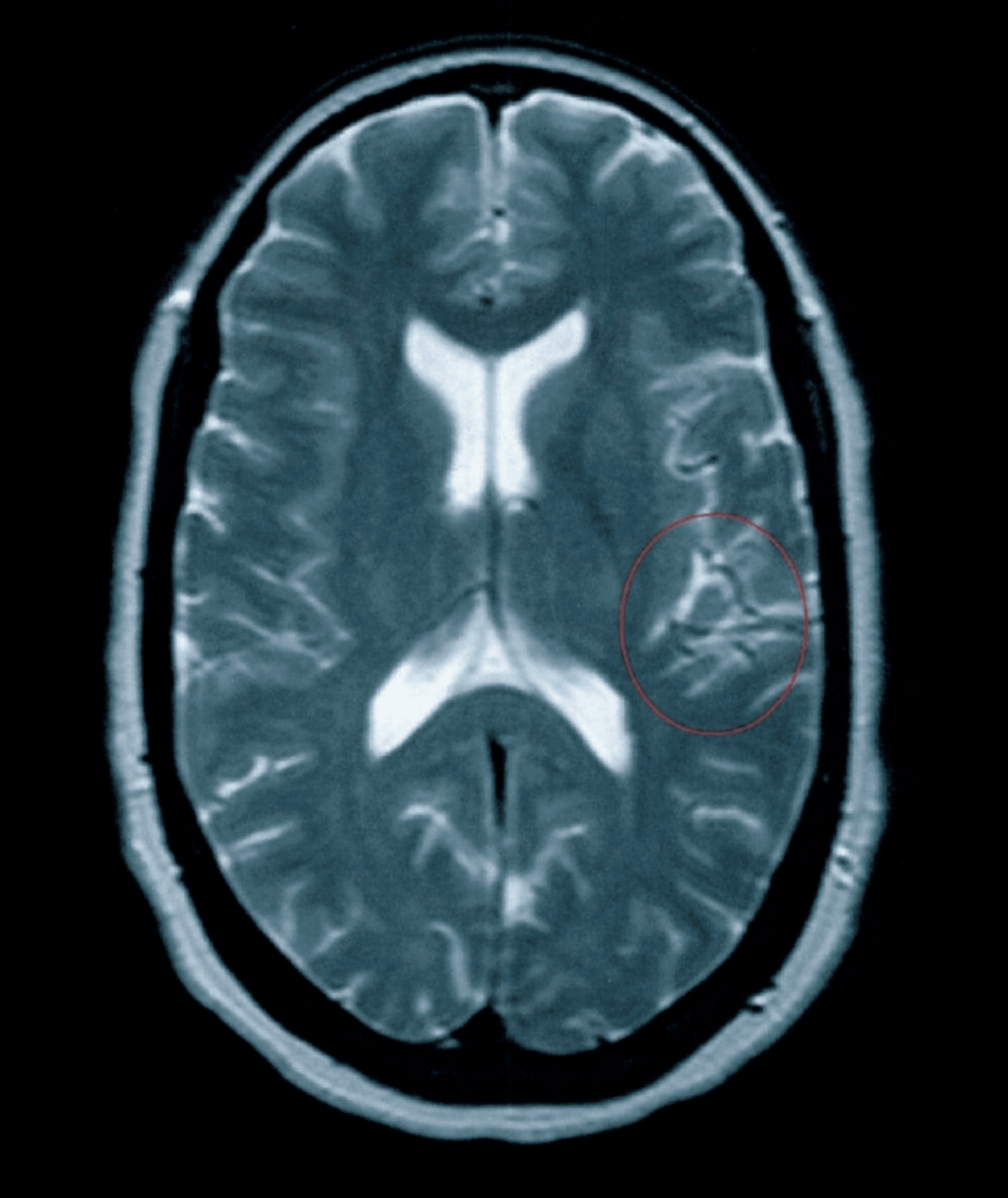
Post-resection MRI of the brain from April 2018 The surgical area is marked with a circle.

Her meningioma was detected in 2015 (Figure 1) and was resected the following year (Figure 2). Her other medical history included hyperlipidemia, major depression, osteoarthritis, mitral valve prolapse, and low back pain, which have persisted throughout the course of her follow-up. Her medications included pravastatin 10 mg daily, multivitamins daily, and Vitamin E 400 units daily. She was offered antidepressants by her primary care provider as well as her psychiatrist, but she refused. She participated in supportive psychotherapy between 2016 and 2018.

The patient underwent neuropsychological testing in 2016 (three months after the surgery) and 2018 (Table 1). Results of cognitive testing administered in 2016 were notable for deficits on tasks of simple auditory attention, short-term verbal memory, and abstraction and adaptive learning. Furthermore, she was noted to have problems with poor adjustment, suspicious beliefs, restlessness, passive-aggressiveness, and generalized anxiety. Functionally, she reported some attention difficulties, such as forgetting where she placed her coffee cup. Her providers concluded that her cognitive deficits were likely attributable to her brain tumor and subsequent resection, and indicated that she met diagnostic criteria for mild cognitive impairment (MCI). Her thyroid hormones (thyroid-stimulating hormone (TSH) and free triiodothyronine (T3)) were normal at all visits. However, her parathyroid hormone was high before the resection (102.4 Pg/ml) and normal at the 2018 and 2023 visits (56.6 and 71.8 Pg/ml).

**Table 1:** Cognitive testing results across evaluations Testing scores interpreted using updated American Academy of Clinical Neuropsychology (AACN) descriptors [9]. Note that below average or better indicated intact functioning

Year	IQ	Sensory	Attention	Processing Speed	Language	Visual Spatial	Memory	Executive Functions
2016	Average	Below Average	Below Average	Below Average	Above Average	Below Average	Low-Average	Extremely Low-Average
2018	Average	Below Average	Below Average	Below Average	Extremely Low-Average	Average	Low-Below Average	Below Average-Average
2023	Average	Average	Average	Average	Average	Below Average	Below Average	Average

The follow-up visit in 2018 revealed deficits in auditory comprehension, abstract reasoning, and left-hand gross and fine motor abilities, as well as variability in attention, learning, and processing speed. Her providers noted these results were consistent with left-sided deficits associated with her prior tumor and resection. Emotionally, she demonstrated mild depressive symptoms and suicidal ideations. None of the medications that she was taking at that time were deemed to be contributing to the depressive symptoms or suicidal ideation (pravastatin, Vitamin E, and multivitamins). Providers, again, diagnosed her with MCI but noted that the extent to which her cognitive deficits were attributable to the residual impact of her tumor and subsequent resection versus ongoing emotional and psychosocial stressors was unclear.

During the most recent evaluation in 2023, the patient reported difficulties learning new names, misplacing items, and comprehending information she read; although she noted some improvement in her cognition since her brain tumor resection in 2016, she expressed concerns that her lingering cognitive difficulties were adversely impacting her work as a secretary. She also reported experiencing depressive symptoms, but did not have any concerns regarding functional abilities. On cognitive testing, she demonstrated very modest deficits in delayed verbal memory and notable improvements in information processing speed and attention/concentration. She scored 26/30 on the Montreal Cognitive Assessment Test (MoCA) [10]. She reported good social support and also support from the Veterans Affairs. She felt that her clinical team met her needs and that several services were available to help with her physical, emotional, and spiritual needs. She reported getting a lot of support from the VA weight-loss program, Motivate Obese Veterans Everywhere (MOVE) [11]. She lost 40 lbs intentionally by joining the MOVE program. She had struggled with social isolation and loneliness in the past, but she has been hosting an exchange student to live with her, and she found improvement in her emotional health and decreased social isolation.

Functionally, she reported complete independence for all basic and instrumental activities of daily living. She scored 4/30 on the Functional Assessment Questionnaire [12]. FAQ scores range from 0-30, with higher scores indicating increasing dependence. Given her performance on cognitive testing and minimal functional declines, it was concluded that she no longer met diagnostic criteria for a neurocognitive disorder, and her subjective cognitive difficulties were likely a result of ongoing depression and chronic pain difficulties.

## Discussion

Our patient showed deficits in multiple domains of cognition within one year of her meningioma resection. This is in keeping with published literature. Gondar et al found in their review that within one year of treatment, deficits in neuropsychological functions are common and that the most commonly reported deficits included in memory, attention, executive function, and language [1]. Our patient demonstrated the most prominent problems in memory, attention, and executive function. Given her tumor on the left side, we would have expected to see more language deficits initially, but they were well retained immediately following the resection. However, language deficits were more prominent in the second test, which was about two years after the resection. In the second test, lateralization of the findings was more prominent.

Our patient was also noted to have prominent anxiety symptoms at the initial visit. Incidentally, she had high PTH levels at that visit. High PTH levels have been associated with anxiety and depression in prior reports [13], although levels of greater than 1000 pg/ml are often associated with increased anxiety [14]. In our patient, the levels were 102.4 pg/ml at the initial visit and in the normal range in the follow-up visits.

In the seven years since her tumor resection, the patient demonstrated notable improvements on neuropsychological testing to such an extent that she was found to no longer meet diagnostic criteria for a neurocognitive disorder diagnosis following her 2023 evaluation. Her executive dysfunction, noted in 2018 testing, completely resolved, which was reflected in her clinical independence and ability to live alone. She was noted to be fully independent in activities of daily living (ADL) and instrumental ADL (IADL). Although she continued to endorse depression, which likely accounted for her subjective cognitive complaints, she intentionally lost 40 lbs by joining the VA MOVE program and cultivated a social support system through hosting an exchange student. Her ability to stick with a plan to lose weight and work towards losing 40 lbs is again a strong indicator of improved executive function. Her clinical picture was also consistent with normal attention, language skills, and processing speed, which were all impaired in her perioperative testing. In sum, she demonstrated notable improvements in cognitive and emotional functioning since her 2016 tumor resection, thus displaying the resiliency of the human brain in its ability to recover from significant insult and the important role neuropsychological and psychiatry services can play in this recovery process. 

One of the limitations of the existing literature is the lack of neuropsychological testing, and that the majority of studies are short-term [1,3,4]. Both are strengths of our report, where detailed neuropsychological and clinical evaluations were available at three distinct time points, as far as seven years from the surgery. Our report highlights that long-term follow-up is essential in such cases. Finally, this study highlights the importance of considering the treatable factors, such as mood and social isolation, in the treatment and management of cognitive difficulties, as doing so can have a significant positive impact on a patient’s functioning and quality of life. The limitation of our case report is the use of subjective reports for depression and functioning. Future studies should consider retrospective chart review as a valid methodology to highlight such cases. 

## Conclusions

This case report highlights long-term improvement in multiple cognitive domains seven years after resection of a meningioma. The improvement in memory and executive dysfunction was impressive in this patient to the extent that the prior diagnosis of MCI was reversed to normal cognition. This case report not only provides a much-needed long-term perspective on the topic but also provides detailed neuropsychological and clinical information in an integrated healthcare system. Affective effects of hormonal changes post tumor resection and concomitant medications were discussed. Furthermore, the importance of providing holistic care that includes the provision of mental healthcare, care for chronic diseases, and social connections is highlighted.
